# Platelet aggregation response to cyclooxygenase inhibition and thromboxane receptor antagonism using impedance aggregometry: A pilot study

**DOI:** 10.14814/phy2.70002

**Published:** 2024-08-20

**Authors:** Auni C. Williams, Kat G. Fisher, Lacy M. Alexander, W. Larry Kenney

**Affiliations:** ^1^ Noll Laboratory, Department of Kinesiology The Pennsylvania State University University Park Pennsylvania USA; ^2^ Center for Healthy Aging The Pennsylvania State University University Park Pennsylvania USA

**Keywords:** aspirin, COX inhibition, impedance aggregometry, platelet aggregation, thromboxane receptor inhibition

## Abstract

Impedance aggregometry is an alternative to light transmission aggregometry that allows analysis of platelet function in whole blood samples. We hypothesized (1) impedance aggregometry would produce repeatable results, (2) inhibition of cyclooxygenase with aspirin would attenuate aggregation responses to collagen and abolish the aggregation response to arachidonic acid (AA), and (3) thromboxane receptor antagonism (terutroban) would attenuate the aggregation response to AA. Venous blood was obtained from 11 participants three times separated by at least 2 weeks. One sample followed 7‐day‐aspirin intervention (81 mg once daily; ASA), the others no intervention (control). Aggregation was induced using 1 μg/mL collagen ([col 1]), 5 μg/mL collagen ([col 5]), and 50 mM AA via impedance aggregometry to determine total aggregation (AUC) analyzed for intra‐test repeatability, inter‐test repeatability, intervention (ASA or control), and incubation (saline or terutroban). [col 1] showed high intra‐test (*p* ≤ 0.03 visit 1 and 2) and inter‐test repeatability (*p* < 0.01). [col 5] and AA showed intra‐ ([col 5] *p* < 0.01 visit 1 and 2; AA *p* < 0.001 visit 1 and 2) but not inter‐test repeatability ([col 5] *p* = 0.48; AA *p* = 0.06). ASA attenuated AUC responses to [col 1] (*p* < 0.01), [col 5] (*p* = 0.03), and AA (*p* < 0.01). Terutroban attenuated AUC in response to AA (*p* < 0.01). [col 1] shows sufficient repeatability for longitudinal investigations of platelet function. [col 5] and AA may be used to investigate mechanisms of platelet function and metabolism at a single time point.

## 1 INTRODUCTION

Cardiovascular disease is the leading cause of death in the United States (Tsao et al., 2023). Nearly 40% of adults over 50‐years‐old regularly use aspirin, a non‐selective cyclooxygenase (COX) inhibitor, for primary or secondary prevention of cardiovascular disease and/or adverse cardiovascular events (Whitlock et al., 2016). The COX metabolite thromboxane is important in the pathophysiology of stroke (Szczuko et al., 2021) as well as platelet aggregation which is augmented through a feedforward mechanism via the thromboxane receptor (TP) (Paul et al., 1999).

The efficacy of this aspirin therapy in preventing platelet aggregation is commonly assessed with light transmission aggregometry (LTA). In LTA, absorbance readings are performed on platelet‐rich plasma samples continuously before and after the addition of a pro‐aggregatory reagent, often tested using multiple reagents including arachidonic acid (AA) and collagen. Once the dispersed platelets begin to aggregate, greater light transmission is permitted through the sample. While considered the gold standard in platelet function testing, the isolation of platelet‐rich plasma for this technique risks removing larger platelets and mechanically disturbing those that remain in the sample to be tested. Impedance aggregometry evades this issue by utilizing whole blood samples to analyze platelet aggregation. However, the utility of impedance aggregometry in aiding investigations of integrative physiology is yet under‐researched (Paniccia et al., 2015).

The purpose of this study was to determine the utility of impedance aggregometry in both a longitudinal and a cross‐sectional analysis of platelet function. We evaluated multiple characteristics of platelet function including total, peak, rate of, and time to begin aggregation following the addition of AA or low and high concentrations of collagen to a whole blood sample incubated in either saline or the selective TP antagonist (terutroban). Additionally, these characteristics were examined following 1 week of aspirin therapy (81 mg/day). We hypothesized impedance aggregometry would produce repeatable results in all properties of aggregation and that aspirin therapy would significantly attenuate the aggregation response to collagen and abolish the aggregation response to AA. Additionally, we hypothesized that terutroban incubation would only significantly attenuate the aggregation response to AA.

## 2 METHODS

The Institutional Review Board at The Pennsylvania State University approved all experimental procedures and protocols (IRB # 00014062). Verbal and written informed consent were voluntarily obtained from all participants prior to participation and in accordance with the guidelines set forth by the Declaration of Helsinki.

### 2.1 Participants

All participants were screened by clinical staff which included a physical examination, medical health history questionnaire, and blood chemistry analysis (Chem 24, Quest Diagnostics, Pittsburgh, PA, USA). All participants were free from cardiovascular and hematologic disease and were not using either over the counter or prescription medication with known primary or secondary hematologic or cardiovascular effects (e.g., anticoagulants, non‐steroidal anti‐inflammatory drugs, antihypertensives, etc.). Women participated at any stage of the menstrual cycle. Participants were not tobacco users and were generally healthy. Prior to experiment visits, participants abstained from consumption of alcohol, caffeine, fish oil, garlic, and participation in strenuous exercise for 24 h.

### 2.2 Experimental design

Peripheral whole blood samples (~8.1 mL) were obtained from participants by the same research nurse specialist in a standardized manner using antecubital venipuncture during three visits separated by at least two (except one participant whose control visits were 1 week apart) but not more than 8 weeks. Two visits followed no intervention (control 1 and control 2, chronologically), and one followed 7 days of low dose aspirin (81 mg nightly) intervention from each participant. The order of these visits (control 1, control 2, or aspirin) was randomized. An additional blood sample (4 mL, BD Vacutainer® K2 EDTA, Franklin Lakes, NJ, USA) was taken each visit to determine platelet count (Quest Diagnostics, Pittsburgh, PA, USA). Samples for impedance aggregometry analysis were collected in three 2.7 mL sodium citrate‐lined blood sample tubes (9:1 ratio, BD Vacutainer®, Franklin Lakes, NJ, USA) and analyzed within 3 h following blood draw. Samples were handled carefully to avoid jostling.

### 2.3 Impedance aggregometry

All samples were analyzed using whole blood impedance aggregometry via CHRONO‐LOG® Model 700 Whole Blood/Optical Lumi‐Aggregometer and associated AGGRO/LINK®8 software (Chrono‐log Corporation, Havertown, PA). A siliconized stir bar was placed in each cuvette (1 mL) along with 500 μL isotonic irrigation grade saline buffer to be incubated approximately 5 min at 37°C. Cuvettes were then filled with 500 μL whole blood and incubated an additional ≥5 min at 37°C. Following incubation, cuvettes were transferred to the magnetic stirrer (1200 rpm) well of the aggregometer and a two‐pronged electrode was placed within the cuvette. The electrical resistance between the prongs was continuously recorded and baseline resistance was set at 0% (0 Ω). A coagulation agonist was added to the cuvette (1 μL or 5 μL collagen at 1 mg/mL or 10 μL AA at 50 mM; [col 1], [col 5], AA, respectively; Chrono‐log, product numbers 385 Collagen and 390 AA) to induce adhesion of the coagulated platelets to the electrodes, increasing the electrical resistance. Following addition of the agonist, aggregation proceeded for 6 min. The time to begin aggregation following the addition of the agonist (Lag, s), maximal aggregation (amplitude, Amp; Ω), rate of the aggregation curve (slope), and total aggregation (area under the aggregation curve, AUC; arbitrary units, a.u.) was continuously calculated and final values at the end of the 6 min were reported. The aggregation response (Lag, Amp, Slope, and AUC) to each agonist was tested in duplicate simultaneously each visit (channel 1 and 2).

Additionally, the aggregation response to each agonist was tested at each visit following incubation of 500 μL whole blood in 500 μL of terutroban solution (TRB; 2.67 × 10^−7^ M, dissolved in lactated Ringer's solution, Sigma–Aldrich, Darmstadt, Germany, SML1198) rather than in saline to selectively antagonize TP. The procedures for testing the aggregation response following incubation of whole blood with TRB were identical to those stated above.

### 2.4 Statistical analysis

Intraclass correlation coefficients (ICC; two‐way mixed effects model, single measures) were determined for each outcome (AUC, Amp, Slope, and Lag; IBM SPSS Statistics 9.4) to assess intra‐test repeatability (Visit 1 Channel 1 vs. Visit 1 Channel 2; Visit 2 Channel 1 vs. Visit 2 Channel 2; Aspirin Visit Channel 1 vs. Aspirin Visit Channel 2). Values obtained from both channels were then averaged, and ICC were determined to assess inter‐test repeatability (Visit 1 Avg vs. Visit 2 Avg, Visit 1 TRB vs. Visit 2 TRB). Significance of ICC were determined through *F*‐tests. Values obtained from control visit outcomes were again averaged to be compared with that of the aspirin visit to determine the impact of the aspirin intervention (Control Visit Avg vs. Aspirin Visit) via two‐way repeated measures ANOVA with multiple comparisons (GraphPad Software, SanDiego, CA, USA). The effect of terutroban incubation compared with saline incubation (Control Visit Saline Avg vs. Control Visit TRB Avg; Aspirin Saline Avg vs. Aspirin TRB) on platelet aggregation response to each agonist was analyzed by two‐way repeated measures ANOVA with multiple comparisons (GraphPad). Platelet count by visit was analyzed with one‐way ANOVA (GraphPad). For all measures, *α* was set at 0.05.

## 3 RESULTS

### 3.1 Participant characteristics

Eleven adults participated in this study [6 women, 5 men; 33 (22–66) years]. Participants were generally healthy (BMI 27 ± 3 kg/m^2^; resting MAP 85 ± 4 mmHg) with normal HbA1c (5.0% ± 2.0%) and cholesterol (total cholesterol 167 ± 33 mg/dL; HDL 53 ± 10 mg/dL; LDL 96 ± 33 mg/dL). Platelet counts were within normal ranges (control visit 1: 262 ± 67 thousand/μL, control visit 2: 277 ± 67 thousand/μL, aspirin visit: 255 ± 62 thousand/μL) for all except one participant (control visit 1: 454 thousand/μL, control visit 2: 444 thousand/μL, aspirin visit: 421 thousand/μL), but did not change by visit (*p* = 0.13; no influential data points). Data (mean ± SD), ICC, and related *p*‐values for intra‐ and inter‐test repeatability are shown in Table 1.

**TABLE 1:** Platelet aggregation characteristic values.

	Intra‐test									Inter‐test		
	Control V1			Control V2			ASA					
	C1	C2	ICC	C1	C2	ICC	C1	C2	ICC	V1	V2	ICC
AUC [col 1]	36 (14)	45 (16)	0.569*	41 (14)	42 (13)	0.848*	9 (12)	8 (11)	0.881*	39 (14)	41 (13)	0.790*
AUC [col 5]	58 (15)	57 (14)	0.689*	61 (10)	58 (8)	0.762*	42 (21)	37 (12)	0.516*	58 (13)	60 (8)	0.015
AUC AA	35 (18)	40 (16)	0.912*	49 (15)	49 (17)	0.933*	1 (3)	0 (1)	0.025	37 (14)	49 (15)	0.503
Amp [col 1]	11 (3)	13 (3)	0.267	12 (3)	13 (2)	0.620*	3 (4)	3 (4)	0.838*	12 (3)	13 (2)	0.739*
Amp [col 5]	16 (3)	16 (3)	0.610*	17 (4)	17 (2)	0.577*	20 (4)	15 (4)	0.651*	16 (3)	17 (3)	0.072
Amp AA	8 (4)	10 (4)	0.630*	12 (3)	12 (3)	0.861*	1 (2)	0 (1)	0.096	9 (3)	12 (3)	0.274
Slope [col 1]	9 (4)	11 (3)	0.757*	9 (4)	9 (4)	0.958*	3 (2)	3 (1)	0.789*	9 (3)	9 (4)	0.783*
Slope [col 5]	10 (3)	10 (3)	0.848*	10 (2)	10 (2)	0.894*	6 (2)	6 (2)	0.814*	10 (2)	10 (2)	0.749*
Slope AA	7 (4)	8 (4)	0.874*	9 (4)	9 (4)	0.956*	2 (1)	2 (1)	0.117	7 (3)	9 (4)	0.598*
Lag [col 1]	93 (43)	89 (44)	0.944*	98 (49)	93 (37)	0.875*	147 (93)	190 (94)	0.528*	93 (42)	95 (42)	0.875*
Lag [col 5]	56 (22)	56 (18)	0.897*	57 (18)	59 (17)	0.954*	85 (40)	93 (43)	0.362	56 (20)	58 (17)	0.645*
Lag AA	42 (21)	39 (18)	0.951*	40 (21)	41 (26)	0.954*	103 (146)	24 (80)	0.927*	42 (18)	40 (23)	0.425

*Note*: Data are represented as mean (SD).

Abbreviations: Amp, amplitude; Amp, Ω; ASA, aspirin visit; AUC a.u.; AUC, area under the curve; C1, channel 1; C2, channel 2; ICC, intraclass correlation coefficient; Lag, s; Slope, a.u; V1, visit 1; V2, visit 2.

* *p* < 0.05.

### 3.2 Intra‐test repeatability (by channel)

With the exception of Amp in response to the [col 1] agonist, AUC, Amp, Slope, and Lag all demonstrated moderate to excellent intra‐test repeatability (agreement between channels; Koo & Li, 2016) and met significance in the control visits in response to [col 1], [col 5], and AA. Intra‐test repeatability was less consistent in the aspirin visit where agreement between channels did not meet significance in response to AA in the AUC, Amp, or Slope characteristics, and in response to [col 5] in the Lag characteristics. However, following aspirin therapy, all response characteristics of [col 1], as well as the AUC, Amp, and Slope characteristics of the [col 5], and the Lag characteristic of the AA response met significance.

### 3.3 Inter‐test repeatability (by visit)

Regarding inter‐test repeatability (agreement between visits) following saline incubation, aggregation responses to [col 5] for the AUC and Amp characteristics, as well as AUC, Amp, and Lag characteristics for AA failed to meet significance. For all characteristics of aggregation responses that did meet significance, the inter‐test repeatability was moderate to excellent. The inter‐test repeatability of all platelet aggregation characteristics following TRB incubation (Table 2) failed to meet significance.

**TABLE 2:** Platelet aggregation characteristic values following terutroban incubation.

	Inter‐test		
	V1 versus V2		
	V1	V2	ICC
AUC [col 1]	38 (19)	52 (16)	−0.642
AUC [col 5]	63 (18)	70 (17)	0.325
AUC AA	1 (1)	6 (16)	0.002
Amp [col 1]	12 (5)	16 (3)	−0.505
Amp [col 5]	18 (4)	20 (4)	0.317
Amp AA	0 (1)	2 (4)	§
Slope [col 1]	8 (4)	10 (5)	−0.2
Slope [col 5]	10 (4)	12 (4)	0.456
Slope AA	2 (0)	3 (2)	1
Lag [col 1]	100 (30)	75 (25)	0.272
Lag [col 5]	63 (18)	58 (17)	0.210
Lag AA	25 (76)	102 (140)	§

*Note*: § no variance in at least one characteristic, ICC could not be calculated. Data are represented as mean (SD).

Abbreviations: Amp, amplitude; Amp, Ω; AUC a.u.; AUC, area under the curve; ICC, intraclass correlation coefficient; Lag, s; Slope, a.u; V1, visit 1; V2, visit 2.

### 3.4 Effect of COX inhibition

**FIGURE 1:**
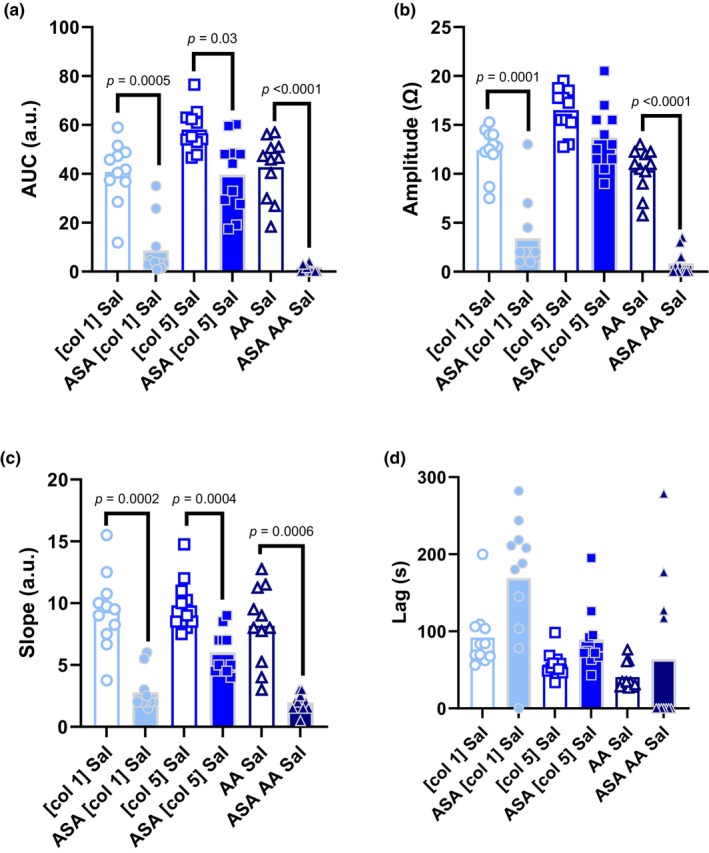
Effect of cyclooxygenase inhibition on platelet aggregation characteristics. (a) AUC, (b) amplitude, (c) slope, and (d) lag in control blood samples (Ctl) and following aspirin therapy (ASA). [col 1], 1 μL collagen; [col 5], 5 μL collagen. Data analyzed by repeated measures two‐way ANOVA with multiple comparisons. AA, arachidonic acid; Sal, saline; TRB, terutroban incubation.

Effects of aspirin therapy are demonstrated in Figure 1. There was a significant main effect of COX inhibition on AUC (*p* < 0.01). Aspirin therapy significantly attenuated the AUC response to [col 1] (*p* < 0.01), [col 5] (*p* = 0.03), and AA (*p* < 0.01). There was a significant main effect of COX inhibition on Amp (*p* < 0.01). Aspirin therapy significantly attenuated the Amp response to [col 1] (*p* < 0.01) and AA (*p* < 0.01), but not [col 5] (*p* = 0.14). There was a significant main effect of COX inhibition on Slope (*p* < 0.01). Aspirin therapy significantly attenuated the Slope response to [col 1], [col 5], and AA (*p* < 0.01 all reagents). There was a significant main effect of COX inhibition on Lag (*p* < 0.01). However, multiple comparisons showed no direct effect of aspirin therapy on the Lag response to [col 1] (*p* = 0.15), [col 5] (*p* = 0.05) or AA (*p* = 0.96).

### 3.5 Effect of thromboxane receptor antagonism

**FIGURE 2:**
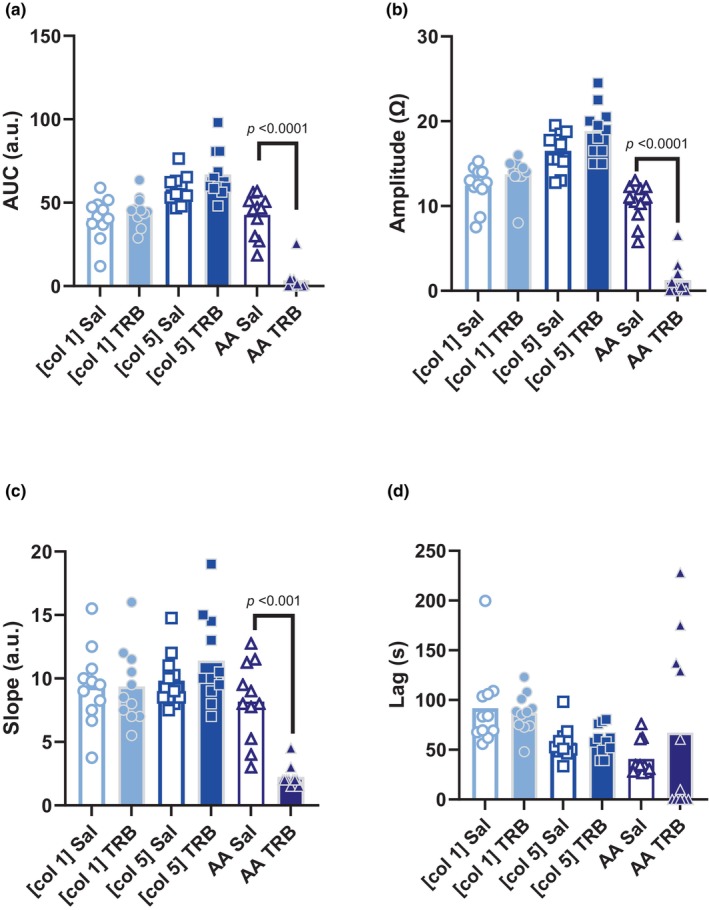
Effect of thromboxane receptor antagonism on platelet aggregation characteristics. (a) AUC, (b) amplitude, (c) slope, and (d) lag following incubation in saline (Sal) or terutroban (TRB). Data analyzed by repeated measures two‐way ANOVA with multiple comparisons.

Effects of TP antagonism with TRB incubation are demonstrated in Figure 2. There was a significant main effect of TRB on AUC (*p* < 0.01). TRB had no effect on the AUC response to [col 1] (*p* = 0.90) or [col 5] (*p* = 0.44) but significantly attenuated the AUC response to AA (*p* < 0.01). There was a significant main effect of TRB on Amp (*p* < 0.01). TRB had no effect on the Amp response to [col 1] (*p* = 0.51) or [col 5] (*p* = 0.20) but significantly attenuated the Amp response to AA (*p* < 0.01). There was a significant main effect of TRB on Slope (*p* = 0.02). TRB had no effect on the Slope response to [col 1] (*p* > 0.99) or [col 5] (*p* = 0.28) but significantly attenuated the Amp response to AA (*p* < 0.01). TRB had no effect on the Lag response to [col 1], [col 5], or AA (main effect *p* = 0.44).

## 4 DISCUSSION

The purpose of the study herein was to determine the repeatability of impedance aggregometry to examine characteristics of platelet aggregation in response to low ([col 1]) and high ([col 5]) concentrations of collagen and AA. Additionally, the influence of aspirin therapy and TP antagonism on platelet aggregation characteristics was subsequently examined. These data suggest that when utilizing impedance aggregometry, low concentration collagen‐induced aggregation demonstrates high intra‐ and inter‐test repeatability, while high concentration collagen and AA induced aggregation primarily demonstrates only intra‐test repeatability. Therefore, low concentration collagen‐induced aggregation may be preferred when examining platelet aggregation characteristics in a longitudinal study design. Additionally, efficacy of COX inhibition with aspirin therapy and TP antagonism with terutroban can be reliably determined using impedance aggregometry.

The mechanisms by which collagen and AA induce platelet aggregation converge around increases in intracellular calcium (Carazo et al., 2024). Following exposure to collagen, phospholipase A and phospholipase C are activated, and phosphatidylinositol 4,5‐bisphosphte is cleaved into inositol 1,4,5‐trisphosphate (IP3) and diacylglycerol (DAG). IP3 increases intracellular calcium by stimulating the release of calcium from the dense tubular system and promotes an influx of calcium from the extracellular milieu (Brass & Joseph, 1985; Roberts et al., 2004). DAG promotes PKC activity, prompting granule secretion and aggregation (Cohen et al., 2011), but additionally stimulates the liberation of AA from the plasma membrane (Hanna & Hafez, 2018). The free AA is metabolized by COX to prostaglandin H_2_, which is further metabolized by thromboxane synthase to produce thromboxane A_2_ (Hanna & Hafez, 2018). TxA_2_ promotes intracellular calcium mobilization as well as platelet shape change and secretion (Paul et al., 1999).

Aggregation induced via moderate or low concentrations of collagen (≤10 μg/mL) are proposed to be mediated in part by synthesis of thromboxane A2, a mechanism which may be proportionally masked by the greater aggregation response to higher concentrations of collagen via PLC pathways mentioned previously (Roberts et al., 2004). This is supported by our findings in that aggregation characteristics in response to 5 μL produced larger AUC, Amp, and shorter lag times compared to 1 μL‐induced aggregation (Figure 1). Additionally, platelet aggregation responses following COX inhibition with aspirin therapy were attenuated to a greater extent in the 1 μL compared to 5 μL collagen agonists, suggesting higher concentrations of collagen induce platelet aggregation through more or different pathways. Therefore, our findings suggest that impedance aggregometry can be used to examine differing mechanisms of platelet aggregation in response to various aggregation agonists.

Following COX inhibition with aspirin therapy, collagen‐induced aggregation at both low and high concentrations demonstrated high intra‐test repeatability, while AA‐induced aggregation shows poor intra‐test repeatability. Inhibition of COX with aspirin therapy significantly attenuated total, peak, and rate of aggregation in response to low dose collagen, high dose collagen, and AA with the single exception of high concentration collagen failing to meet significance in peak aggregation. This further suggests that impedance aggregometry is a sufficient method to examine platelet aggregation characteristics in varying conditions such as COX inhibition.

Subsequently, reliability of impedance aggregometry to investigate the influence of TP antagonism on platelet aggregation was examined. Platelet aggregation following TP antagonism with terutroban incubation demonstrated poor inter‐test repeatability across all characteristics of platelet aggregation in response to low concentration collagen, high concentration collagen, and AA. When values were averaged between visits, TP antagonism with terutroban incubation significantly attenuated the aggregation response to AA but neither concentration of collagen with regard to total, peak, and rate of aggregation. Time to begin aggregation was unaffected by terutroban incubation. AA is able to induce aggregation via COX‐mediated thromboxane A2 production which promotes intracellular calcium mobilization and induces shape change in platelets, and further potentiates aggregation through the release of dense granules containing many other pro‐aggregatory agents (Paul et al., 1999). The present study extends these findings by demonstrating no direct involvement of the TP in collagen‐induced aggregation but a significant role for the receptor in aggregation induced from AA (Figure 2). Utilizing impedance aggregometry to examine multiple pro‐aggregatory reagents in investigations of mechanisms of platelet function may provide valuable insight into multiple aggregation pathways.

LTA is the current reference method for platelet function analysis. We do not intend to diminish the benefits of analyzing the specific function of a cell type in near isolation, but the effect of the extracellular environment on platelet aggregation, as described above, indicates more translatable findings may be obtained by allowing platelets to remain in whole blood samples during analysis. Additionally, given the multiple pathways by which mechanical disturbances may impact platelet activity (Mammadova‐Bach et al., 2023), it may be preferable to minimize physical manipulation of platelets prior to analysis.

## 5 LIMITATIONS

The key limitation of this study is the low sample size in comparison to other investigations of platelet function and given the variability of aggregation characteristic values. However, the sample size utilized in this investigation is comparable to that of other integrative physiology research, and as such provides important findings on an additional tool to aid in investigations of human physiology. Another potential limitation of this work is that this sample was primarily young and healthy. Whether these findings can be extended to an older or disease population remains to be confirmed.

## 6 CONCLUSIONS

Our findings suggest that impedance aggregometry can be used to examine platelet aggregation characteristics in response to multiple aggregation inducing reagents in differing conditions. Low concentration collagen may be a pro‐aggregatory stimulus with sufficient repeatability to allow for longitudinal investigations of platelet function. High concentration collagen‐induced aggregation instigates a greater degree of aggregation than low concentration collagen and may be an effective tool to investigate mechanisms of platelet function and metabolism at a single time point. Similarly, AA is a potent aggregatory agent that may be utilized to investigate mechanisms of platelet function and metabolism at a single time point. Therefore, impedance aggregometry may be used as an alternative or secondary approach to LTA that increases translatability and reduces technique‐induced error.

## AUTHOR CONTRIBUTIONS

A.C.W., K.G.F., L.M.A., and W.L.K. conceived and designed the research; A.C.W. and K.G.F. performed the experiments; A.C.W. analyzed the data, A.C.W. and K.G.F. interpreted the results of the experiments; A.C.W. prepared the figures; A.C.W. drafted the manuscript; A.C.W., K.G.F., L.M.A., and W.L.K. edited and revised the manuscript; A.C.W., K.G.F., L.M.A., and W.L.K. approved the final version of manuscript.

## FUNDING INFORMATION

This work was supported by NIH Grant R01 AG067471 (to W. L. Kenney), NIH F31 HL170616‐0 (to A. C. Williams), and NIH R01 HL161000 (to L. M. Alexander).

## CONFLICT OF INTEREST STATEMENT

No conflicts of interest, financial or otherwise, are declared by the authors. Lacy M. Alexander is on the editorial board for *the American Journal of Physiology—Heart and Circulatory Physiology* and is the editor in chief for *the Journal of Applied Physiology*. W. Larry Kenney is an editor of *Journal of Applied Physiology*. Neither WLK nor LMA were involved or had access to information regarding the peer‐review process or final disposition of this article. An alternative editor oversaw the peer‐review and decision‐making process for this article.

## Data Availability

Data will be made available upon reasonable request.
